# Recent Progress in Functional-Nucleic-Acid-Based Fluorescent Fiber-Optic Evanescent Wave Biosensors

**DOI:** 10.3390/bios13040425

**Published:** 2023-03-27

**Authors:** Zheng Wang, Xinhui Lou

**Affiliations:** Department of Chemistry, Capital Normal University, Xisanhuan North Road. 105, Beijing 100048, China

**Keywords:** functional nucleic acid, evanescent wave biosensors, optical fiber, aptamers, DNAzymes

## Abstract

Biosensors capable of onsite and continuous detection of environmental and food pollutants and biomarkers are highly desired, but only a few sensing platforms meet the “2-SAR” requirements (sensitivity, specificity, affordability, automation, rapidity, and reusability). A fiber optic evanescent wave (FOEW) sensor is an attractive type of portable device that has the advantages of high sensitivity, low cost, good reusability, and long-term stability. By utilizing functional nucleic acids (FNAs) such as aptamers, DNAzymes, and rational designed nucleic acid probes as specific recognition ligands, the FOEW sensor has been demonstrated to be a general sensing platform for the onsite and continuous detection of various targets ranging from small molecules and heavy metal ions to proteins, nucleic acids, and pathogens. In this review, we cover the progress of the fluorescent FNA-based FOEW biosensor since its first report in 1995. We focus on the chemical modification of the optical fiber and the sensing mechanisms for the five above-mentioned types of targets. The challenges and prospects on the isolation of high-quality aptamers, reagent-free detection, long-term stability under application conditions, and high throughput are also included in this review to highlight the future trends for the development of FOEW biosensors capable of onsite and continuous detection.

## 1. Introduction

Onsite continuous detection techniques are highly desired for real-time monitoring of pollutants in environmental waters [1] and food production processes [2,3]. They also are of central importance for the development of wearable medical devices to routinely measure biomarkers [4,5]. These technical advances are dramatically changing traditional lab-based strategies into more and more convenient, automatic, and labor-free means. To realize onsite continuous detection for daily use, a technology should be sensitive, specific, affordable, automated, rapid, and reusable, criteria referred to as “2-SAR”. Numerous biosensors have been reported in recent years with the overall purpose to achieve high sensitivity or rapidity. Quite often, expensive and complicated signal amplification strategies, most commonly enzymatic reactions, are used to enhance the detection sensitivity. In addition, tedious sample processing steps prior to detection are required to avoid matrix interference. These offline processes greatly lengthen the assay time and complicate the operation, preventing their application for continuous detection, where the timely monitoring of the concentration of the target is required. Ideally, the detection should be reagent-free, where no reagents need to be added for signal amplification or sensor regeneration.

Of the existing sensing platforms, only a few meet the “2-SAR” criteria and are commercially available [6]. The most outstanding example is the wearable electrochemical sensor for the real-time detection of blood sugar levels [7,8]. The continuous detection is based on the electrochemical signals generated by the in situ oxidation reactions of glucose catalyzed by the enzymatic electrode. The technique is elegant, but only suitable for targets that can undergo specific redox reactions. Most pollutants or biomarkers do not have redox activity under mild conditions and the specific enzymes are also not available. Moreover, the sensitivity of this technique is in the low millimole per liter (mM) concentration range, which does not meet the sensitivity requirements for most pollutants and biomarkers (typically in the picomole to nanomole per liter concentration range). Electrochemical devices are also commercially available for the onsite detection of heavy metal ions [9]. They are based on the redox reaction of metal ions, rendering high sensitivity and specificity. The limitation is the interference from the sample matrix resulting from the nonspecific absorption of various components on the electrode, which interferes with the current. Therefore, offline sample pretreatment is commonly required to ensure the reusability of the electrodes, leading to the long assay time and the high cost. Fluorescent evanescent wave sensors are another type of portable optical sensors that are suitable for continuous detection [6,10,11,12,13]. They are based on the evanescent wave generated on the surface of waveguide materials (chip or fiber) upon laser incidence from the light-dense medium to the light-sparse medium. Planar waveguide sensors are capable of the simultaneous detection of multiplex targets but require a much more sophisticated and expensive optical system than fiber optical evanescent wave (FOEW) sensors [14]. Fluorescent FOEW sensors are, therefore, more suitable for continuous detection merely from an affordability point of view. In fact, fluorescent FOEW sensors have attracted more attention than planar waveguide sensors in recent years. Over the past half-century, tremendous advances have been made in FOEW sensors, from the optical system, fluid system, to the modification chemistry of optical fiber and sensing mechanisms [6,10,11,12,13].

Functional nucleic acids (FNAs) include aptamers, DNAzymes, and rationally designed nucleic acids, which all have the ability to specifically recognize targets and are widely used in various types of biosensors [15]. Due to rapid advances in FNAs in the last thirty years, the application scope of FOEW sensors has been rapidly expanding from common water quality parameters including pH, temperature, oxygen, ions, refractive index, and (dissolved) gases and vapors to almost any type of target. FNA-FOEW sensors have been reported for the detection of all types of targets including metal ions, small molecules, proteins, nucleic acids, bacteria, and viruses with the promise of more affordable, durable, and flexible detection compared to antibody-based FOEW sensors. The sensitivity and dynamic range of all FNA-based sensors are strongly affected by the probe density on the sensor surface [16]. FNA-based electrochemical sensors are typically orders of magnitude more sensitive than FOEW, but they are difficult to regenerate. In contrast, FNA-FOEW sensors can be regenerated, enabling repeated measurements using the same sensor and, therefore, a higher accuracy.

After over 40 years of innovation, fluorescent FOEW biosensors are proceeding quickly and have found respective applications in highly diversified fields. Several excellent reviews on them have been published with different focuses on instrument configurations and patents [6], enzyme- or antibody-based biosensors [10,14], or a specific type of target such as viruses [17]. Recently, Loyez et al. published an excellent review on aptamer-based optical fiber sensors using all types of sensing strategies including surface plasmon resonance (SPR), localized SPR (LSPR), lossy mode resonance, fluorescence, and others [18]. The review covers a broad scope with less specific information for certain types of sensing strategy. There is no review with a focus on the functional-nucleic-acid-based fluorescent FOEW (FNA-FOEW) for diverse types of targets. In view of the significant difference of FNAs from antibodies, a comprehensive review of FNA-FOEW sensors would be a valuable complement to the reviews of fiber-optic sensing technology. The novelty of our review is to summarize the recent progress, challenges, and perspectives of FNA-FOEW sensors with a special focus on the probe immobilization chemistry and diverse sensing mechanisms for different types of targets. Please note that this review does not include SPR- or silicon-photonic-based FOEW biosensors. In those biosensors, the detection of targets is achieved by measuring the refractive index changes, instead of fluorescence intensity changes [19,20,21].

**Figure 1:**
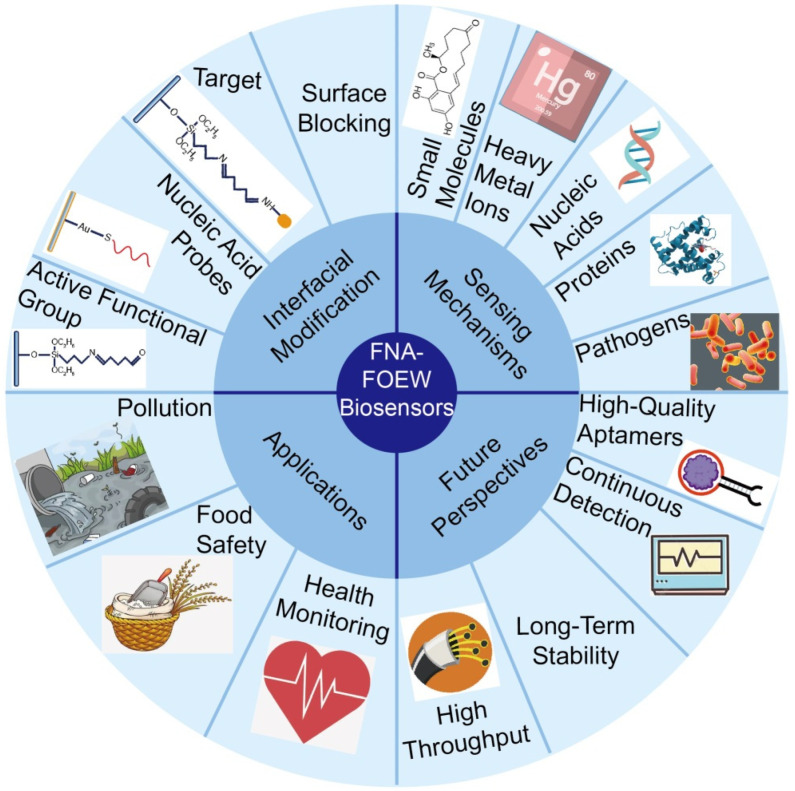
Scheme diagram of the content of this review.

The major content of this review is summarized in Figure 1. Since the sensor optical and fluidic system is general for the optical fibers with different coating materials, the above-mentioned reviews and many other books and articles are excellent resources for detailed information. This review focuses on the surface chemistry of the optical fiber and the sensing mechanisms, and the optical physics are briefly introduced. We started with an introduction of the recent advances in FNAs, which are responsible for specific target recognition in FNA-FOEW sensors. We then conducted a brief introduction of the major components of FOEW sensors and optical mechanisms for real-time fluorescence detection. The major methods for fiber modification and the representative sensing mechanisms for different types of targets are systemically reviewed according to the surface chemistry of the fiber. The challenges and prospects of fluorescent FNA-FOEW sensors are provided at the end of this review.

## 2. Functional Nucleic Acids (FNAs)

**Figure 2:**
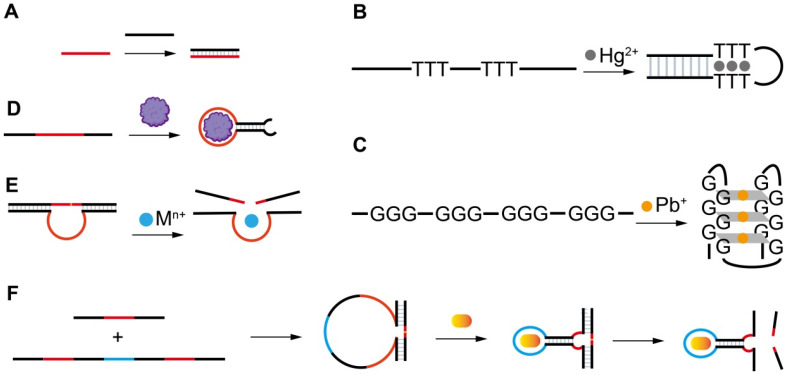
The artificial FNAs most popularly used in biosensors including FNA-FOEW sensors. (**A**) Complementary DNA probe (cDNA); (**B**) rationally designed thymine (T)-rich probe for highly specific Hg^2+^ detection; (**C**) rationally designed guanine (G)-rich probe for highly specific Pb^2+^ detection; (**D**) aptamers; (**E**) DNAzymes specific to various metal ions; (**F**) aptazymes for nonmetal ion targets.

Nucleic acids were initially defined as a hereditary substance that carries genetic information. Later, research clearly evidenced that some nucleic acids, called FNAs, also play many other important biological functions in vivo or in vitro [15,22,23,24,25]. FNAs with catalytic activity or specific molecular recognition capability have attracted tremendous attention in many fields. They are divided into two categories: natural and artificial FNAs. Natural FNAs are found in vivo and include ribozymes and riboswitches. Artificial FNAs are obtained by in vitro screening or engineering, and include rationally designed nucleic acid probes, aptamers, DNAzymes, and aptazymes (Figure 2). Artificial FNAs are attractive synthetic probes because of their low cost, ease of synthesis and modification, high stability, and biocompatibility. They have been extensively used for sensing detection [26,27], drug delivery [28] and disease therapy [22], molecular imaging [29], and self-assembly of nanomaterials [30,31]. Among these applications, their applications in fluorescent, colorimetric, and electrochemical sensors are dominant and have shown the greatest potentials for practical uses.

The different types of FNAs enable the specific recognition of different types of targets. The first groups of FNAs are short complementary DNA probes (cDNAs) (Figure 2A), typically 10–25 mer in length, which are rationally designed to be completely or partially complementary to the target DNA or RNA sequences. The hybridization reactions between cDNAs and target sequences cause the formation of duplex structures in a solution or on the sensor surface, allowing the specific detection of target sequences by various signal transformation means. The hybridization reaction has also been extended for the indirect detection of other types of targets beyond nucleic acids by incorporating the following FNAs. Another type of rationally designed FNA sequence is metal-ion-specific DNA probes. The most applied ones are thymine (T)-rich probes highly specific to Hg^2+^ [32,33,34] (Figure 2B) and guanine (G)-rich probes highly specific to Pb^2+^ [35,36,37,38] (Figure 2C). The two types of probes respectively undergo a large conformation change from random coil to duplex or G-quadruplex structures upon binding to Hg^2+^ or Pb^2+^. Rationally designed probes have also been reported for other metal ions, such as Ag^+^ [39] and K^+^ [40,41], but are much less applied because they are not the most common pollutants of concern in the environment or food.

Both aptamers and DNAzymes are in vitro isolated nucleic acids from a nucleic acid library by SELEX (Systematic Evolution of Ligands by Exponential Enrichment) technology [27,42,43]. Aptamers are specific binding ligands without catalytic activity (Figure 2D). Since the invention of SELEX, more than one thousand aptamers have been isolated, targeting all types of targets from metal ions, small molecules, and proteins to complex targets such as bacteria and viruses [44,45,46,47]. The specific binding event can be signally transformed in diverse sensing platforms. DNAzymes are FNAs with diverse enzymatic activities. DNAzymes with cleavage activity catalyzed by a wide spectrum of metal ions as cofactors have been reported in recent years [27,48] (Figure 2E). Among them, the most popularly used are those specific to Pb^2+^. The DNAzyme forms two duplex segments with its substrate, leaving the central loop region as the metal ion recognition pocket. In the presence of the right metal ion, the cleavage activity is activated due to the binding between the metal ion and the loop, and the phosphodiester bond next to the RNA nucleotide in the substrate sequence is cleaved. The catalytic cleavage of the substrate sequence lays the basis for various sensing mechanisms. Due to their catalytic property, one metal ion can trigger multiple cycles of cleavage, leading to high sensitivity and negating the use of protein enzymes for signal amplification. 

Aptazymes are a type of FNA with both catalytic activity and specific binding capability, in which the binding pocket in DNAzymes for metal ions is designed through engineering to include aptamer sequences [49] (Figure 2F). Even though the design is elegant, tedious optimization is required and their extended applications are limited. So far, no aptazymes have been used in FOEW sensors.

In FOEW sensors, the above-described FNAs are either functionalized on the fiber surface or added into the test sample. The fluorescent group is typically modified on the FNAs or their complementary sequences for signal transduction of the specific molecular recognition event that occur on the fiber surface. The representative designs are illustrated in the following sections.

## 3. The Major Components of Fluorescent FOEW Sensors and Optical Mechanisms for Real-Time Fluorescence Detection

**Figure 3:**
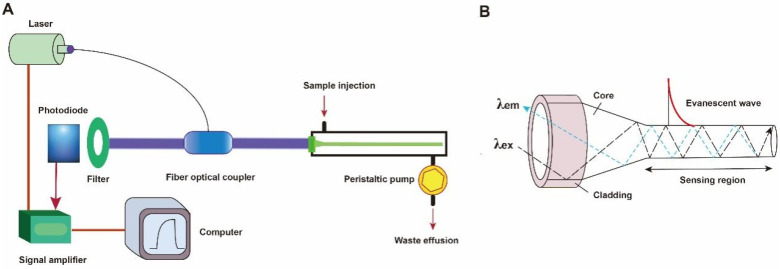
(**A**) The components of a typical fluorescent FOEW sensor; (**B**) the structure of the tapered fiber and the evanescent wave generated vertical to the fiber surface.

A typical fluorescent FOEW sensor consists of an optical system (laser, optical fiber coupler, filter, photodiode, and signal amplifier), a mechanic fluidic system (peristaltic pump, tubes, reaction chamber), and a data analysis system (software and computer) (Figure 3A) [6]. The optical fiber is installed inside the reaction chamber. The inlet and outlet tubes are connected to the chamber for automatic sample injection and waste elution with controlled flow rate and time [50]. For the purpose of portability, the FOEW sensors have been miniaturized and several portable FOEW sensors have been commercialized for in situ applications [10]. Continuous onsite measurements are realized by the regeneration of the sensor surface by simply rinsing with sodium dodecyl sulfate solution (0.5% SDS, pH 1.9) after each measurement [51]. Very recently, the miniaturized all-fiber-optical system and microfluidic system have been integrated with smartphones for onsite real-time quantitative detection of bisphenol A and norfloxacin in 15 min with high sensitivity and reusability, and automated interpretation of reporting results [52].

**Figure 4:**
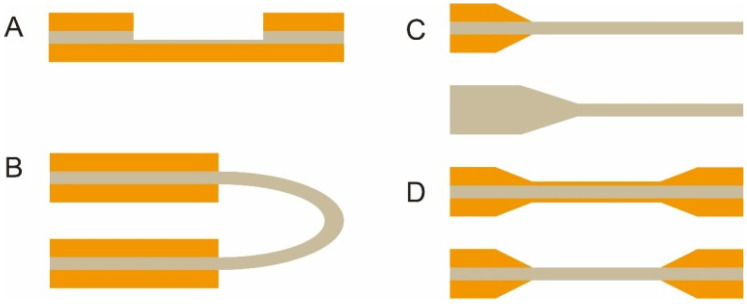
The most common optical fiber structures. (**A**) D-shaped fibers; (**B**) U-shaped fibers; (**C**) tapered fibers; (**D**) biconical fibers. The gray part is the core, and the orange part is the cladding. The section without the cladding is the sensing region, where the surface modification is typically performed prior to the sensing.

The optical fiber is the core element of an FOEW sensor for real-time fluorescence sensing. It usually consists of the inside core and the outside cladding. The refractive index (RI) of the fiber core is higher than that of the fiber cladding. Light travels through the fiber via total internal reflection. The material of the core is usually transparent glass (SiO_2_) or plastic with excellent laser transport capability and ease of surface modification. With the purpose of improving sensitivity, optical fibers with diverse shapes have been reported. They can be roughly divided into two categories, fiber gratings (FGs) and structured optical fibers (SOFs) [11]. FGs are optical fibers that have periodic gratings, which change the refractive index (RI) of the core. Common SOFs include D-shaped, U-shaped, tapered, and biconical fibers (Figure 4). The SOFs, especially the tapered fiber, are much easier to fabricate than FGs. Optical fibers for the preparation of tapered fibers are commercially available at quite a low price (approximately USD 1–2 per probe). For this reason, tapered fibers are the most widely used fibers in FOEW sensors. 

Taking the tapered fiber as an example, the generation of the optical phenomenon inside and on the surface of the fiber was illustrated (Figure 3B). For other shapes of optical fibers, an excellent review has introduced them in detail [11]. When the laser incidence from the light-dense medium (such as SiO_2_) to the light-sparse medium (such as an aqueous sample) and the angle of incidence is greater than the critical angle, the refracted light disappears, and total reflection occurs. The total reflection of the incident laser inside the fiber leads to the formation of the evanescent wave field propagated vertical to the surface of the fiber. The distance is called the depth of penetration (d_p_) when the intensity of the evanescent wave decays to 1/e of the original light wave intensity [53]. The d_p_ is a function of several parameters as shown in the following equation and is typically in the 100–200 nm range.

$$d_{p}=\frac{\lambda }{2\pi {\left(n_{1}^{2}\sin^{2}\theta -n_{2}^{2}\right)}^{1/2}}$$

where λ represents the wavelength of the incident light; n_1_ represents the refractive index of the fiber core medium; n_2_ represents the refractive index of the surrounding solution medium; and θ is the angle of incidence of the fiber at the interface. A large d_p_ is the key to achieve high sensitivity for FOEW sensors. The tapering, launch angle, and taper length of the fiber strongly affect the sensitivity and can be easily optimized.

The evanescent wave excites the fluorescence emission of fluorophores inside the evanescent wave field. The intensity of the emission fluorescence is then real-time measured by the photodiode detector after the filter. It is worth pointing out that FOEW sensors tend to show a higher signal-to-noise (or lower background interference) ratio compared to the fluorescence measurements conducted in solution because only the fluorophores within the evanescent wave field can be effectively excited [6].

In order to realize the simultaneous detection of multiple targets, Long et al. designed and manufactured a compact dual-color FOEW sensor installed with two lasers for the simultaneous excitation of two fluorophores at distinct wavelengths. The simultaneous detections of aflatoxin M1 (AFM1) and ochratoxin A (OTA) [54], *Escherichia coli* (*E. coli*) O157:H7 and *Salmonella typhimurium* [55], or acetamiprid and fipronil [56] were respectively achieved. The group further simplified the optical structure, where a single-multi mode fiber optic coupler was employed to replace the sophisticated confocal optical system for the transmission of two excitation lights and dual-color fluorescence [57,58]. A photodiode detector was used instead of a photomultiplier for the simultaneous detection of dual-color fluorescence.

Besides the solid fibers described above, a hollow-core fiber has also been used in fluorescent FNA-FOEW sensors [59]. Wang et al. recently demonstrated that hollow-core microstructured antiresonant fibers (HARFs) can stringently confine light in the fiber core, ensuring a high signal and sensitivity. The hollow-hole fiber or capillary serving as a waveguide was first invented in 2000 by Liger et al., and has been commercialized. Those seeking more information on the progress of the systems using the hollow-hole fiber can refer to the excellent review [10].

## 4. Optical Fiber Interfacial Modification Methods

Optical fibers can be chemically modified at the interface before use or left unmodified, depending on the different sensing mechanisms. Unmodified optical fibers have been used for the detection of diverse types of targets including bacteria and metal ions [60]. Taking advantage of the large size of bacteria (at least submicron in diameter) and the short evanescent wave depth (typically less than 100 nm), an unmodified bare SiO_2_ fiber has been used for the detection of *E. coli* O157:H7. A bare fiber etched with a nanoporous layer was used and better sensitivity was achieved, which not only separates *E. coli* O157:H7 from the aptamer, but also enhances the performance of the evanescent wave [55,61]. The benefits for using the unmodified fiber include no need for surface modification, easy surface regeneration, and low cost. However, the sensitivity is commonly much lower than those using modified fibers. To overcome the sensitivity limitation, offline signal amplifications are required to improve the detection sensitivity.

The well-controlled surface modification of fiber optics is critical for sensor performance including sensitivity, specificity, dynamic range, binding kinetics, long-term stability, reproducibility, and surface regeneration. Many different chemical reactions have been used for surface modification. Among them, the silanization and the mild crosslinking reactions compatible with aqueous solutions are the most popularly used. In the following, we summarized the most popularly used methods for the interfacial modification of SiO_2_ fibers according to different purposes including modification to introduce active functional groups, nucleic acid probes, and targets, or to block the surface.

### 4.1. Modification to Introduce Active Functional Groups on Fiber Surface

**Figure 5:**
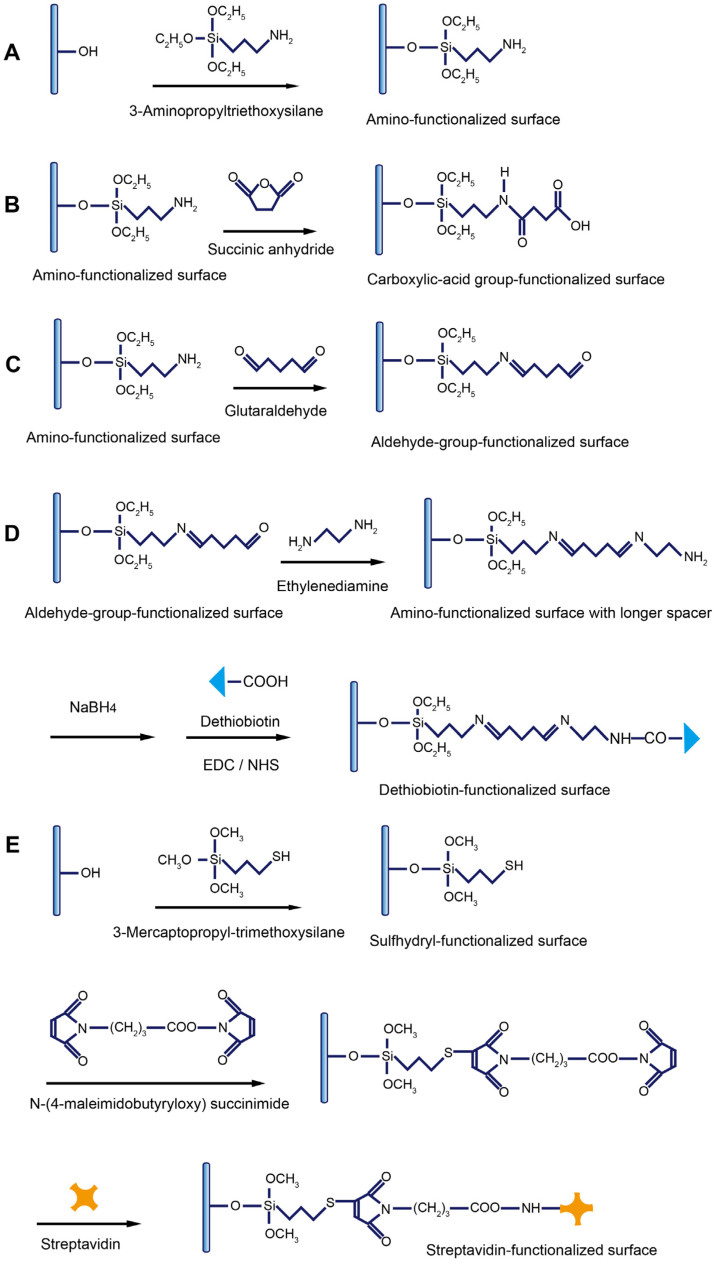
The representative methods to modify the fiber with active functional groups: (**A**) amino-group-functionalized surface; (**B**) carboxylic-acid-group-functionalized surface; (**C**) aldehyde-group-functionalized surface; (**D**) dethiobiotin-group-functionalized surface; and (**E**) sulfhydryl, NHS ester, or streptavidin-group-functionalized surface. EDC: 1-ethyl-3-(3-dimethylaminopropyl)carbodiimide; NHS: N-hydroxysuccinimide.

Through silanization reactions and the following one- or two-step crosslinking reactions, various functional groups can be facilely introduced on the SiO_2_ fiber surface. The most popularly used silanization reaction is the reaction between the hydroxyl groups and 3-aminopropyltriethoxysilane (APTS), by which the fiber is functionalized with amino groups (Figure 5A) [62]. The hydroxyl groups are generated by soaking the fiber in piranha solution.

The amino-group-modified fiber surface can be further converted into other active function groups such as the carboxylic acid group (Figure 5B) and aldehyde group (Figure 5C) by soaking the fiber in different crosslinkers. The crosslinker with dual-amino groups is commonly used to lengthen the spacer (Figure 5D). Very recently, a single-stranded binding protein (SSB) was immobilized on the fiber surface using the same EDC/NHS-mediated reaction for the capture of the free aptamer [63]. The dethiobiotin groups bind to streptavidin or streptavidin-modified molecules with weaker affinity than biotin, rendering easier interruption of the binding for surface regeneration [64,65,66]. They can be modified on the fiber through the highly efficient coupling reaction between the amino group on the fiber and the ECD/NHS-activated carboxylic acid group on dethiobiotin (Figure 5D). Very recently, a single-stranded binding protein (SSB) was immobilized on the fiber surface using the same EDC/NHS-mediated reaction for the capture of the free aptamer [63]. By using 3-mercaptopropyl-trimethoxysilane (MTS), the sulfhydryl groups are introduced to the fiber surface [67]. One maleimide group in the bifunctional crosslinking reagent N-(4-maleimidobuty-ryloxy) succinimide (GMBS) reacts specifically with sulfhydryl groups and forms a stable thioether linkage. The other maleimide group further reacts with the amino group in streptavidin to covalently immobilize streptavidin on the fiber surface (Figure 5E). Streptavidin can be used for immobilization of any target labeled with the biotin group via the strong binding between streptavidin and biotin [63].

### 4.2. Immobilization of Nucleic Acid Probes on Fiber Surface

**Figure 6:**
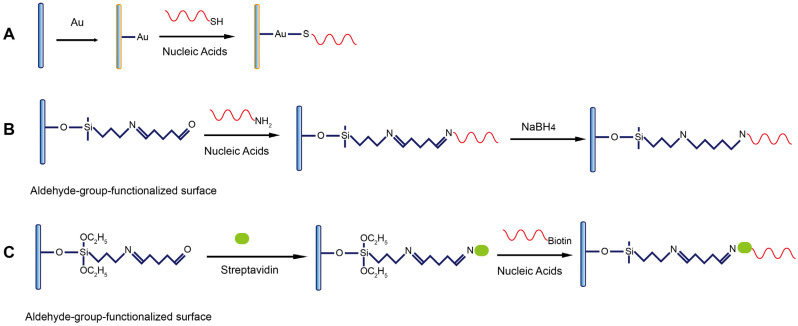
The representative methods to functionalize the fiber with nucleic acids modified with (**A**) thiol; (**B**) amino; and (**C**) biotin groups.

Three types of immobilization chemistry are used for the immobilization of nucleic acid probes on the fiber including gold-thiol (Au-S) coordinate interaction (Figure 6A), covalent coupling (Figure 6B), and noncovalent binding (Figure 6C). To be compatible with these modification strategies, the nucleic acid probes are respectively modified with the thiol, amino, or biotin group at either end of the terminals.

The coating of a thin layer of gold film is typically used to enhance the evanescent wave and, therefore, the sensing sensitivity in various types of waveguide sensors including FOEW sensors, but is not often used in fluorescent FOEW sensors. Gold nanoparticles are a universal fluorescence quencher and widely used in biosensors. They have been recently used in FNA-FOEW sensors for the detection of exosomes in blood samples from breast cancer patients [56]. The gold film is compatible with several commonly used surface characterization techniques such as SPR [65], quartz crystal microbalance (QCM) [66], and electrochemical sensors [67]. Thus, multiple characterization techniques can be simultaneously used to investigate the complicated surface effects on sensor performance. The thiolated nucleic acids are the most popularly used for the immobilization on the gold surface due to the well-controlled formation of the self-assembled monolayer. However, the Au-S bond is prone to be auto-oxidized in air, resulting in quite a limited surface stability (typically within one to two weeks) [68,69]. Therefore, the Au-S-based nucleic acid immobilization method is more suitable for theoretical study, but not robust enough for practical uses.

The immobilization of nucleic acid probes via the covalent bond can overcome the stability limitation of the Au-S-based immobilization strategy. Our experiments showed that fibers functionalized with DNA probes retain stable hybridization signals over several months when they are stored at 4 ºC in a humid environment. The high stability has also been demonstrated by other research groups [68,69,70]. The drawback of this covalent immobilization method is its multiple-step process and the difficulty to quantitatively monitor each modification step on the surface. The most popularly used route consists of several steps: (1) oxidation to form hydroxyl groups, (2) silanization to convert the hydroxyl groups into an amino group, (3) the transformation of the amino to an aldehyde group by amine aldehyde condensation reaction, (4) covalently coupling the amino-modified nucleic acid probes, and (5) reduction of the less-stable imine bond into a C-N single bond and the unreacted aldehyde groups into hydroxyl groups by NaBH_4_ reduction [50]. Many works use a similar approach to immobilize amino aptamers on the fiber surface with slight differences [51,71,72,73,74,75,76,77,78]. For examples, glycine [60,79] and NaCNBH_3_ [60,80] were used to deplete the remaining aldehyde sites after the coupling of aptamers. A similar route has also been used for the attachment of nucleic acid probes on PMMA fibers [81].

Another strategy for nucleic acid probe immobilization is via the strong noncovalent binding between biotin and streptavidin. The whole process is largely the same as the covalent route described above except for the final noncovalent binding step, where the biotin-labeled nucleic acid probes are attached to the surface via the strong noncovalent binding between streptavidin (or avidin) and biotin [82,83,84]. The advantage of the use of noncovalent binding to immobilize the nucleic acid probes might be its better antifouling property. However, the long-term surface stability of the sensor is not as good as the covalent one, besides the high cost of streptavidin. Therefore, this immobilization strategy is not often used for continuous detection.

### 4.3. Immobilization of Target Molecules

**Figure 7:**
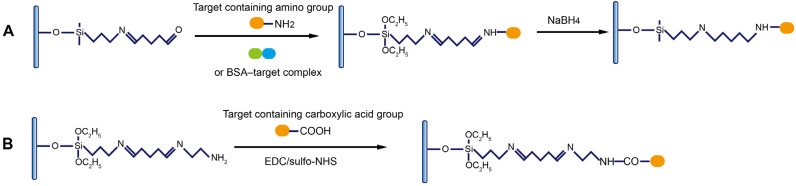
The representative methods to modify the fiber with targets containing (**A**) amino or (**B**) carboxylic acid groups.

The immobilization of targets (most commonly small molecules and their protein complexes) on the fiber is most widely performed when antibodies are used as recognition elements. Thus, the targets in the testing samples and those bound on the surface competitively bind with the antibodies added in the testing samples, enabling the signal-off detection of targets. In FNA-FOEW sensors, targets, complementary probes to FNAs, and FNAs all can be immobilized on the fiber surface for different sensing mechanisms. The following two processes are most utilized to immobilize the targets according to their available function groups on the optical fiber. For a target with at least one primary amino group, such as kanamycin [50], it is covalently immobilized on the fiber through the crosslinking reaction between the aldehyde group pre-functionalized on the fiber and the amino group of the target (Figure 7A). This method is also used for the immobilization of the target–protein complex when a target itself has no amino group. For example, to construct an FOEW sensor for the detection of 17β-estradiol, a complex of 17β-estradiol and bovine serum albumin (BSA) was immobilized on the surface of an optical fiber via the reaction of an amino group of BSA with an aldehyde group on the fiber surface [85]. For a target with at least one carboxylic acid group, such as dethiobiotin (Figure 5D) and ochratoxin A [86], the carboxylic acid group is activated by EDC/sulfo-NHS or NHS in solution, followed by the highly efficient reaction between the activated carboxyl group with the amino group on the fiber.

### 4.4. Surface Blocking of Optical Fiber

After the immobilization of nucleic acid probes or targets, it is necessary to block the fiber surface to quench the remaining active groups or to minimize the nonspecific absorption of fluorescent probes and other components in the sample matrix. Quenching the unreacted active function groups is easy. For example, the remaining aldehyde groups can be depleted by the small-molecule reagents containing the amino group, such as Tris or ethanolamine. The minimization of the nonspecific absorption on the sensor surface remains one of the most challenging issues for biosensors, including FNA-FOEW sensors. In recent years, several types of antifouling reagents have been reported, such as thiolated oligo(ethylene glycol) [87,88,89], mercaptohexanol [90], zwitterionic moieties [91,92], polyethylene glycol [93], and BSA [94]. The oligo(ethylene glycol) moiety, zwitterionic moieties, and BSA are well known for their capability to prevent the nonspecific absorption of proteins. Mercaptohexanol can prevent the nonspecific absorption of nucleic acids [90]. Unfortunately, the most widely reported thiolated passivation reagents are only used for the gold surface, and are not compatible with the SiO_2_ fiber, where the well-controlled self-assembly of the passivation monolayer is formed by Au-S interactions. BSA has been used to block all types of surfaces ranging from microplates, nitrocellulose membranes, to electrodes and glass surfaces. So far, BSA is the most popularly used blocking agent in FNA-FOEW sensors due to its low cost and chemical compatibility [60,66,75,80,85,86].

Even though the specific mechanisms are unclear, the antifouling capability of the oligo(ethylene glycol) moiety and zwitterionic moieties has been related to the formation of an aqueous layer [87,88,89,91,92,93]. Quite differently, the mechanism of BSA for the minimization of nonspecific absorption is due to the physical coverage of the active binding sites of the surfaces. Therefore, the complete coating of the surface is essential. One important advance is the formation of the passivation layer using denatured BSA at pH near its isoelectric point (4.6), instead of the routine neutral pH. At the isoelectric point, the BSA is neutral, allowing the formation of a more uniform passivation layer on the fiber. The iep-BSA-blocked FNA-FOEW sensor showed a 10-fold-improved limit of detection (LOD, S/N = 3, 125 pM) for the detection of target DNA and the highest number of regeneration cycles (120 cycles) [95]. Even though the BSA layer minimized the nonspecific absorption of nucleic acids, its capability to resist the interference from the complex sample matrix is still not satisfactory.

## 5. Sensing Mechanisms

**Figure 8:**
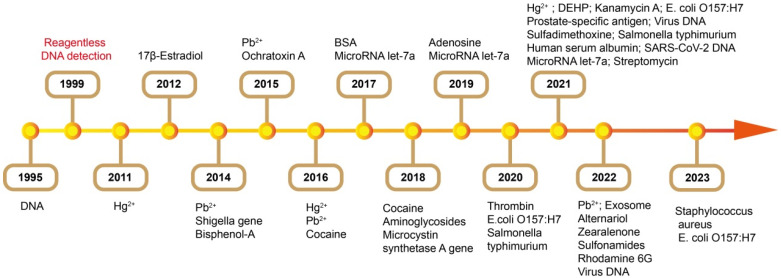
The major progress of FNA-FOEW sensors according to the target scope.

FNA-FOEW sensors have been utilized for the detection of all types of targets after Krull et al. demonstrated their capability for the detection of DNA in 1995 (Figure 8). In the following, we summarized the progress of the sensing mechanisms according to the types of targets. We drew the core sensing mechanisms in the literature in the same style to highlight the differences among different strategies. We also summarized the important progresses in the tables to facilitate the direct comparison of the performance (LOD, dynamic range, reusability, et al.) of each method for the detection of small molecules, heavy metal ions, and nucleic acids, respectively (Table 1, Table 2 and Table 3).

### 5.1. Separation-Free Detection of Small Molecules Using Aptamers

**Figure 9:**
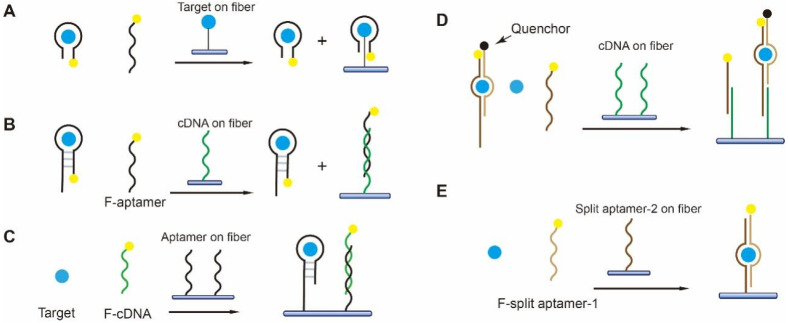
The separation-free detection of small-molecule targets using FNA-FOEW sensors installed with fibers functionalized by (**A**) target [85,86]; (**B**) cDNA [75]; (**C**) aptamer [71,72,77]; (**D**) cDNA complementary to one of the split aptamer fragments [73]; or (**E**) one fragment of the split aptamer [76,80].

Small molecules are the most diverse type of environmental and food pollutants including antibiotics, toxins, hormones, etc. In recent years, the number of small-molecule-binding aptamers has been rapidly increasing, which lays the basis for the development of aptamer-based FOEW sensors. Different from antibodies, aptamers not only bind with their targets, but also bind with the complementary sequences. In addition, some aptamers, called split aptamers, can be split into two fragments, which are assembled upon target binding [96]. Thus, aptamer-based sensing mechanisms for the detection of small-molecule targets are more diverse than the antibody-based ones (Figure 9). We classified the current reported methods into the separation-free and offline-separation-based methods. According to the different surface modifications of the fiber, we divided the first category methods into five different sensing mechanisms.

**Figure 10:**
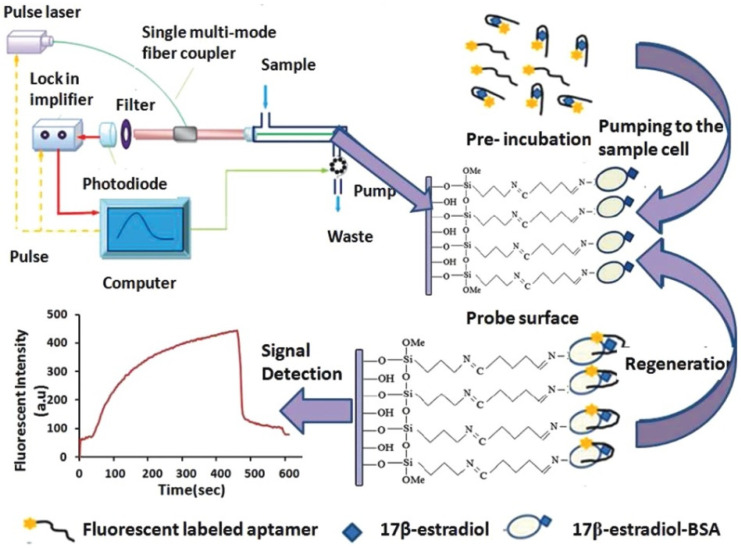
Aptamer-based FOEW sensor for rapid and sensitive detection of 17β-estradiol in water samples. Reprinted from Yildirim et al. (2012) [85], Copyright (2018), with permission from American Chemical Society.

The first aptamer-based FOEW sensor for small molecule detection was reported in 2012 for the detection of 17β-estradiol, a frequently detected endocrine-disrupting compound (EDC) in environmental waters (Figure 10) [85]. The sensing mechanism is an analogue of the most popularly used, indirect competitive binding immunoassays for small-molecule targets, in which 17β-estradiol 6-(o-carboxy-methyl)oxime-BSA is covalently immobilized on the surface (Figure 9A and Figure 10). The 17β-estradiol molecules in the water sample and immobilized on the surface competitively bind with the fluorophore-labeled aptamer in the water sample, enabling the signal-off detection of 17β-estradiol in the water sample with an LOD of 2.1 nM. The same strategy was also used for the development of an FOEW sensor for the detection of mycotoxin ochratoxin A (OTA, LOD = 0.97 nM) in wheat samples [86]. The advantage of this type of sensing method is high accuracy because the dynamic range is typically only within two orders of magnitude. The sensors also possessed excellent reusability (>100 times) and quite a short assay time (within 10 min). The limitations included low sensitivity and the difficulty of target immobilization for the targets lack of functional groups.

To avoid the complication of target immobilization, the cDNA was immobilized on the fiber instead of the target (Figure 9B) for the detection of bisphenol A (BPA) with an LOD of 1.86 nM [75]. BPA is a known endocrine disruptor and one of the most serious environmental contaminants. The sensors retain excellent reusability (>100 times) and quite a short assay time (within 10 min). However, the LODs are typically in the low nanomole per liter (nM) concentration range for the targets when the dissociation constants (K_D_) of their aptamers are 10–100 nM (Table 1). The sensitivity is not high enough for practical applications because the samples have to be diluted to minimize the matrix interference.

**Table 1:** Detection of small molecules.

Target	Sensing Mechanism	LOD (nM)	Linear Range (nM)	Real Sample	Reusability (Times)	Time ^a^ (min)	Selectivity	Ref.
Bisphenol A	Figure 9B	1.86	2–100	Wastewater	100	10	Estriol; 17β-estradiol; 2,4-dichlorophenol; bromophenol blue; phenol; phenol red	[75]
Ochratoxin A	Figure 10A	3	6–500	Oat samples	300	5	Aflatoxin B1; aflatoxin B2; deoxynivalenol; chloramphenicol	[66]
Ochratoxin A	Figure 9A	0.97	1.81–31.0	Wheat sample	100	10 ^b^	Aflatoxin B1; deoxynivalenol	[86]
Cocaine	Figure 9D	165.2	200–2 × 10^5^	Human serum	40	7.5 ^b^	Kanamycin; amikacin; sulfadimethoxine; ibuprofen	[73]
Aminoglycoside ^c^	Figure 10C	26	0–1 × 10^3^	Milk	60	N/A	Tetracycline; terramycin; chlortetracycline; ibuprofen; bisphenol A; sulfadimethoxine	[78]
Cocaine	Figure 9B	1.05 × 10^4^	1 × 10^4^–5 × 10^6^	N/A	50	16.5	Neomycin; sulfadimethoxine; ampicillin; kanamycin	[74]
Adenosine	Figure 9E	2.5 × 10^4^	5 × 10^4^–3.5 × 10^6^	N/A	N/A	N/A	N/A	[76]
Streptomycin	Figure 9D	33	60–526	Waters ^d^	100	5	Penicillin G; tetracycline; tobramycin; neomycin; kanamycin A	[80]
Zearalenone	Figure 9C	2.31 × 10^−6^	1 × 10^−6^–0.1	Corn flour extract	28	6	Deoxynivalenol; aflatoxin B1, B2, G1, G2, M1; ochratoxin A; fumonisin B1, B2	[77]
Sulfonamides	Figure 9C	0.2 × 10^−6 e^ 0.5 × 10^−6 f^ 4.8 × 10^−3 g^	1 × 10^−7^–1 × 10^−3 e^ 1 × 10^−7^–1 × 10^−2 f^ 1 × 10^−3^–10 ^g^	Lake water	40	5	Kanamycin A; ampicillin; doxycycline; diethylhexyl phthalate; tobramycin	[71]
Alternariol	Figure 9C	42 × 10^−6 h^ 6 × 10^−6 i^ 2 × 10^−6 j^	1 × 10^−4^–0.1 ^h^ 1 × 10^−5^–1 × 10^−2 i^ 1 × 10^−6^–0.1 ^j^	Wheat powder	35	5	Vomitoxin; zearalenone; patulin; altenuene; tenuazonic acid; tentoxin	[72]

^a^ Detection time only; ^b^ including incubation time; ^c^ kanamycin A, kanamycin B, amikacin, gentamycin; ^d^ mineral spring water, bottled purified water, tap water, surface water, wastewater; ^e^ for sulfaguanidine; ^f^ for sulfamethizole; ^g^ for sulfamethoxazole; ^h^ original truncated aptamer; ^i^ bivalent aptamer; ^j^ trivalent aptamer.

**Figure 11:**
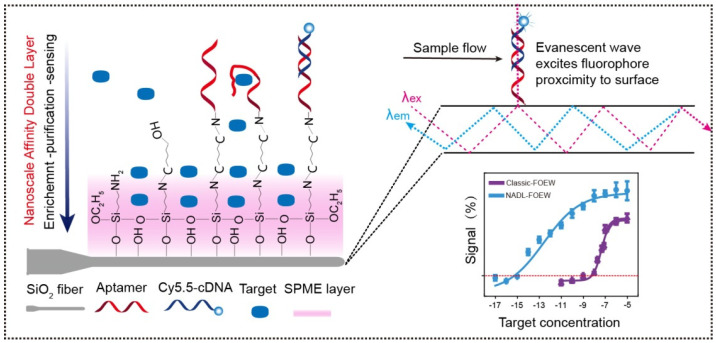
NADL-FOEW for rapid and ultrasensitive detection of small-molecule targets in environmental water, fresh milk, wine, and urine samples. Reprinted from Zhao et al. (2021) [50], Copyright (2021), with permission from American Chemical Society.

Recently, our group devised an ultrasensitive FNA-FOEW sensing platform to overcome the sensitivity limitation, named the nanoscale affinity double layer (NADL)-FOEW sensor, for the detection of small-molecule targets regardless of their hydrophobicity (Figure 11) [50]. In the NADL-FOEW sensor, the aptamer is covalently immobilized on the fiber along with a nonspecifically absorbed Tween 80 thin layer (Figure 9C and Figure 11). The fluorophore-labeled cDNA and target competitively bind with the immobilized aptamer, enabling the signal-off detection of targets. Different from the target or cDNA-immobilized strategies, the NADL-FOEW sensor enables in situ target enrichment, purification, and sensing on the same surface simultaneously, therefore dramatically improving the LOD down to the unprecedented femtomolar ranges. So far, we have demonstrated the ultrasensitive and specific detection of kanamycin [50], sulfadimethoxine [50], di-(2-ethylhexyl) phthalate [50], zearalenone [77], sulfonamides [71], and alternariol [72] in diverse matrices including environmental water, fresh milk, wine, wheat, and corn extracts. Both the detection sensitivity and the dynamic range all meet the practical applications. The reusability (around 30 times for different targets) is not as good as the FOEW sensors shown in Figure 9A,B, but is much higher than typical electrochemical biosensors (commonly less than three times). We also demonstrated the first class-specific detection of sulfonamides, where the total concentration of 14 sulfonamides spiked in the environmental water was continuously detected [71]. Due to the rapid increase in the numbers of synthetic structure analogues, the total concentration of structure analogues is commonly used for the evaluation of pollution levels. This is the first example to demonstrate the applicability of the FOEW sensor for the detection of the total concentration of multiple structure analogues. The broad dynamic range is a benefit for the detection of targets with a broad concentration distribution in real samples such as industrial waste. However, it also limits applications for the detection of targets, such as immunosuppressive drugs, with a narrow effective concentration range. This is an important issue. 

Enzymatic signal amplification has also been used to improve the sensitivity of FNA-FOEW sensors. Li et al. reported a portable chemiluminescent FNA-FOEW sensor for the ultrasensitive onsite detection of four mycotoxins in food samples. The SSB was immobilized on the fiber surface and the unbound biotin-labeled free aptamer was captured by the SSB, while the target-bound aptamer was not captured by the SSB. The streptavidin–biotinylated horseradish peroxidase (SA-Bio-HRP) complex was then employed as a signal amplification tag to catalyze the oxidation of luminol into a chemiluminescent product [63]. The sensors possess low LODs in the range of 0.015–0.423 pg/mL for different mycotoxins. The limitation of this method is the high cost since both SSB and SA-Bio-HRP are expensive. The reusability of the sensor would also be limited since SSB is protein, which tends to be denatured.

Split aptamers enable novel sensing mechanisms that are not suitable for monolithic aptamers. He et al. reported the first split-aptamer-based FOEW sensor for the detection of cocaine in serum, in which the cDNA complementary to one fragment of the split aptamer was immobilized on the fiber (Figure 9D). The flow sample contains cocaine and the two split aptamer fragments, one labeled with fluorophore and the other labeled with a quencher. The binding between cocaine and the pair of fragments not only allows the fluorescence quenching, but also prohibits the hybridization of the fluorophore-labeled fragment with the cDNA. Thus, only the free fluorophore-labeled fragment hybridizes with the cDNA immobilized on the fiber, enabling the signal-off detection of cocaine (LOD = 165.2 nM) [73]. The advantage of this sensing mechanism is that it is a separation-free process. The limitations of this design include its low sensitivity and the signal-off detection mode. 

Later on, a signal-on sensing mechanism was reported by immobilizing one of the split aptamers on the fiber (Figure 9E). The fluorophore-labeled single fragment of the spilt aptamer and the target, adenosine, are assembled with the other fragment of the split aptamer attached to the surface, enabling the detection of adenosine (LOD = 25 μM) [76]. The same sandwich strategy has also been used for the continuous detection of streptomycin (LOD = 25 μM) [80]. The signal-on detection is attractive, while the split aptamers sacrifice binding affinity compared to the original aptamer, resulting in unsatisfactory sensitivity.

### 5.2. Offline-Separation-Based Detection of Small Molecules Using Aptamers

**Figure 12:**
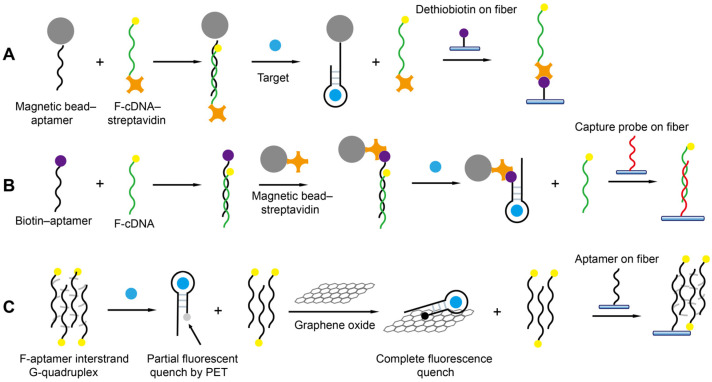
The offline-separation-based detection of small-molecule targets using FOEW sensors installed with fibers functionalized by (**A**) dethiobiotin [66]; (**B**) capture probe complementary to cDNA of aptamer [74]; and (**C**) aptamer [78].

Offline separation using magnetic beads or graphene oxide has been coupled with FOEW sensors for the detection of small molecules (Figure 12). It not only helps to minimize the matrix interference on the fiber, but also enables the new surface regeneration method [66], signal-on detection [66,74], or novel sensing mechanisms [78]. The limitation is a relatively longer assay time and less convenient operation compared to the separation-free strategies described above.

The first offline magnetic-bead-based detection strategy was reported by Shi et al. for the signal-on detection of OTA (LOD = 3 nM) [66]. In their method, the OTA-binding aptamer is immobilized on the magnetic beads and the cDNA probe is dual-labeled with fluorophore and streptavidin. The cDNA is immobilized on the magnetic beads via the hybridization with the aptamer. The cDNA probe is displaced from the magnetic bead when OTA binds with the aptamer. The released cDNA in the supernatant after magnetic separation is captured by dethiobiotin functionalized on the fiber via the strong dethiobiotin–streptavidin interaction. The sensor can be easily regenerated over 300 times without losing sensitivity. Later, the same group used a similar strategy for the signal-on detection of cocaine (LOD = 10.5 μM) [74]. The major difference is the use of a fiber coated with the capture probe complementary to the fluorophore-labeled cDNA to quantitate the amount of replaced cDNA via hybridization.

Graphene oxide is an effective fluorescence-quenching material and a widely used absorption material. Taking advantage of its selective absorption capability to single-stranded DNA over G-quadruplex, He et al. used graphene oxide as both quencher and separation matrix to realize the continuous detection of aminoglycoside antibiotics with an LOD of 26 nM [78]. Specifically, the fluorophore-labeled aptamers fold into an interstrand G-quadruplex in the absence of aminoglycosides. The fluorescence is partially quenched due to the photo-induced electron transfer (PET) when the aptamer binds with aminoglycoside. The fluorescence of the aptamer–aminoglycoside complex is further quenched by graphene oxide when it absorbs on graphene oxide. The remaining free aptamer forms the G-quadruplex with the surface-attached aptamer, enabling the signal-off detection of several aminoglycosides. The limitation of this sensing mechanism is its limited generality. Only the aptamers that can form multiple strand complexes may possibly be compatible with this sensing mechanism.

### 5.3. Detection of Heavy Metal Ions Using DNAzyme and T-Rich Probes

Heavy metal ions are extremely harmful to both the ecosystem and human health. The sensitive and specific detection of heavy metal ions in the environment and food is of paramount importance. The rapid development of FNAs specific to certain metal ions has provided valuable chances for the development of biosensors. Even though numerous FNA-based biosensors have been reported in recent years, most of them are only suitable for a one-time test and not capable of continuous detection [97,98]. Several FNA-FOEW sensors have been reported for the onsite continuous detection of Pb^2+^ and Hg^2+^, which show great potential for real applications (Table 2).

**Figure 13:**
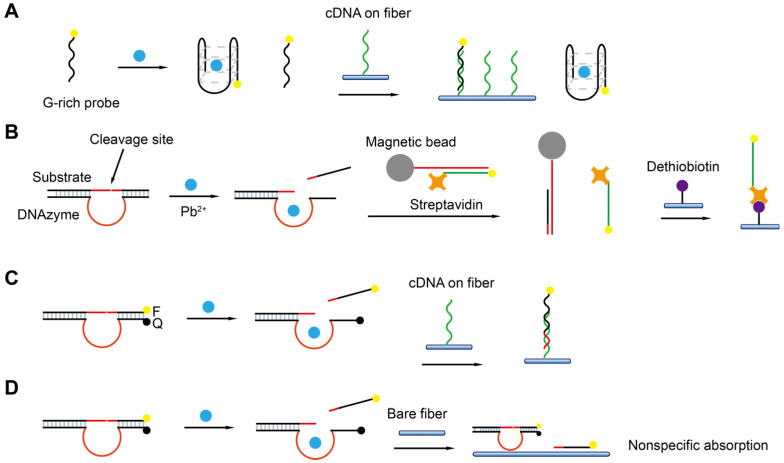
Schematic diagrams of FOEW sensors for Pb^2+^ detection using (**A**) G-rich probe [99] or DNAzymes via (**B**) offline magnetic-separation-based strategy [65] and (**C**,**D**) separation-free means enabled by cDNA functionalized fiber [79] or bare fiber [100].

The first detection of Pb^2+^ using FNA-FOEW was reported by Long et al. in 2014 [99]. They used the fluorophore-labeled G-rich aptamer as a molecular probe (Figure 2C), which forms a stabilized G-quadruplex structure upon binding with Pb^2+^. The G-quadruplex cannot hybridize with the cDNA attached to the fiber, enabling the signal-off detection of Pb^2+^ (Figure 13A). The good regeneration of the sensor in the absence of Pb^2+^ was demonstrated (50 times). The sensing mechanism is simple; however, the sensor may suffer from difficulty in surface regeneration after Pb^2+^ detection due to the nonspecific binding of Pb^2+^ on the fiber.

Later, Shi et al. reported the first DNAzyme-FOEW sensor for the detection of Pb^2+^ with a greatly improved reusability (>300 times) and long-term stability (over one month at room temperature) [65]. The Pb^2+^-specific DNAzyme 8–17 is so far the most popularly used DNAzyme in all types of Pb^2+^ biosensors [101]. In their method, the sensor was constructed using DNAzyme 8–17 in an offline-separation-based mode (Figure 13B). The magnetic beads are immobilized with the duplexes, which are formed by the streptavidin and fluorophore dual-modified signal probe and the strand complementary to the substrate of 8–17. Pb^2+^ catalyzes the cleavage of the substrate of 8–17 and the released fragment hybridizes with the above complementary strand on the magnetic bead. The dual-labeled signal probe is replaced and subsequently detected by the dethiobiotin-modified fiber. The signal-on detection of Pb^2+^ was then realized with an LOD of 1 nM and the linear range was from 20 nM to 800 nM. The limitation of this method is the requirement of offline operation (1 h), which complicates the detection process and lengthens the total detection time.

The DNAzyme 8–17 and GR-5 were respectively used to construct the separation-free FOEW sensors by direct detection of the fluorophore-labeled cleaved substrate (LOD = 20 nM) either using the cDNA functionalized fiber (Figure 13C) [79] or the bare fiber (Figure 13D) [100]. The hybridization and the nonspecific absorption were respectively utilized to capture the cleaved substrate. LODs of 20 nM and 9.34 nM were respectively achieved by both methods. The detection time was similar for both sensors (approximately 13 min per test). The use of bare fiber for the detection of Pb^2+^ provides several advantages including low cost and better reusability. The nonspecific absorption-based sensing methods are prone to be strongly affected by the presence of nontargets. Therefore, the limitation is the hard-to-predict matrix interference from different samples.

**Table 2:** Detection of heavy metal ions.

Target	Sensing Mechanism	LOD (nM)	Linear Range (nM)	Real Sample	Reusability (Times)	Time (min)	Selectivity	Ref.
Pb^2+^	Figure 13A	0.22	1–300	Bottled water; tap water; lake water; wastewater	50	10	Hg^2+^ Ni^2+^ Co^2+^ Cd^2+^ Ca^2+^ Cu^2+^ Fe^3+^ Ag^+^ K^+^	[99]
Pb^2+^	Figure 13B	1	20–800	Bottled water; tap water; mineral spring water	250	60 + 5 ^a^	Hg^2+^ Ni^2+^ Co^2+^ Cd^2+^ Ca^2+^ Cu^2+^	[65]
Pb^2+^	Figure 13C	20	0–1 × 10^4^	Dan Jiang Kou reservoir water	18	13	Ag^+^ Ca^2+^ Zn^2+^ Fe^2+^ Cu^2+^ Cd^2+^ Co^2+^ Mn^2+^ Mg^2+^ Pb^2+^ Hg^2+^ Fe^3+^ Al^3+^	[79]
Pb^2+^	Figure 13D	9.34	N/A	Tap water; underground water; bottled purified water; human serum	N/A	13	Zn^2+^ Mg^2+^ Ca^2+^ Cu^2+^ Cd^2+^ Hg^2+^	[100]
Hg^2+^	Figure 14C	2.2 × 10^−2^	2.2 × 10^−2^–10	Dan Jiang Kou reservoir water	18	7	Ag^+^ Ca^2+^ Zn^2+^ Fe^2+^ Cu^2+^ Cd^2+^ Co^2+^ Mn^2+^ Mg^2+^ Pb^2+^ Hg^2+^ Fe^3+^ Al^3+^	[79]
Hg^2+^	Figure 14A	2.1	N/A	Tap water; pinery wastewater plant; bottled water	100	6	Ca^2+^ Zn^2+^ Fe^2+^ Cu^2+^ Sn^2+^ Cr^2+^ Mn^2+^ Ni^2+^ Pb^2+^	[51]
Hg^2+^	Figure 14B	1.06	75–1 × 10^3^	Bottled water; tap water; pond water	200	N/A	Ni^2+^ Co^2+^ Cd^2+^ Pb^2+^ Ca^2+^	[64]
Hg^2+^	Figure 14D	1	7–1200	Effluent of wastewater treatment plants	31	30	Ni^2+^ Co^2+^ Cd^2+^ Pb^2+^ Ca^2+^ Mg^2+^	[60]
Hg^2+^	Figure 14E	8.5	N/A	Tap water; bottled water; lake water; underground water	N/A	10	Ca^2+^ Zn^2+^ Cu^2+^ Mg^2+^ Cd^2+^ Pb^2+^	[60]

^a^: offline incubation time + online detection time.

**Figure 14:**
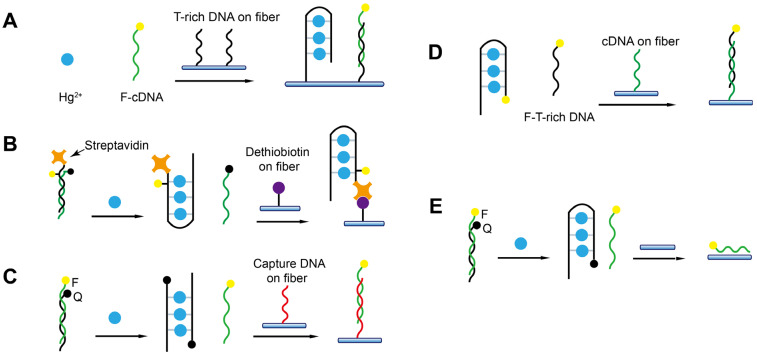
Schematic diagrams of T-rich probe-based FOEW sensors for Hg^2+^ detection using fibers functionalized by (**A**) T-rich DNA [51]; (**B**) dethiobiotin [64]; (**C**) capture DNA [79]; (**D**) cDNA [60]; or (**E**) nothing (bare fiber) [60].

The T-rich probes have been widely used for the specific detection of Hg^2+^ [102] since the finding of the specific coordination binding between T-Hg^2+^-T [32]. Five different strategies have been developed to realize the detection of Hg^2+^ and are all in the separation-free modes (Figure 14). The sensing mechanisms are very similar to those used for the detection of Pb^2+^ except the molecular probes are different. The advantages and limitations of each method are not repeatedly discussed. The first Hg^2+^-FOEW sensor was reported in 2011 by A.Z. Gu et al. (Figure 14A) [51]. In their method, the T-rich probe is immobilized on the optical fibers. Hg^2+^ and the fluorophore-labeled cDNA probe compete for the T-rich sequence on the fiber, realizing the signal-off detection of Hg^2+^ (LOD = 2.1 nM). The sensing mechanism is simple, but the signal-on sensing mechanism is preferred, and the sensitivity is not high enough to meet the requirements.

Later, a signal-on method was developed by adopting a strand-displacement-based method with slightly improved sensitivity (LOD = 1.06 nM), where the T-rich probe is dual-labeled with fluorophore and streptavidin and the cDNA is labeled with a quencher (Figure 14B). The cDNA and the T-rich probe are separated upon the binding of Hg^2+^ with the T-rich probe. The released T-rich probe is then captured by the dethiobiotin-coated fiber, enabling the signal-on detection of Hg^2+^ [64]. The LOD was further improved to 22 pM when a different pair of T-rich probe and cDNA are used, and they are respectively labeled with quencher and fluorophore (Figure 14C). The displaced cDNA upon the binding of Hg^2+^ with T-rich probe is detected by the capture probe immobilized on the fiber [79].

The sensing mechanism was further simplified by only using the fluorophore-labeled T-rich probe (LOD = 1 nM) (Figure 14D) [60]. The T-rich probe forms a hairpin structure upon binding to Hg^2+^ and is unable to bind to the cDNA on the fiber. The sensing mechanism was also simplified by using a bare fiber and the quencher-labeled T-rich probe/fluorophore-labeled cDNA pair (LOD = 8.5 nM) (Figure 14E) [60]. The signal-on detection of Hg^2+^ is realized by the fluorescence increase caused by the adsorption of the released cDNA on the bare fiber. The method avoids the surface modification of the fiber, while the limitation is its lower sensitivity compared to the above modified-fiber-based methods. 

### 5.4. Detection of Nucleic Acids

**Figure 15:**
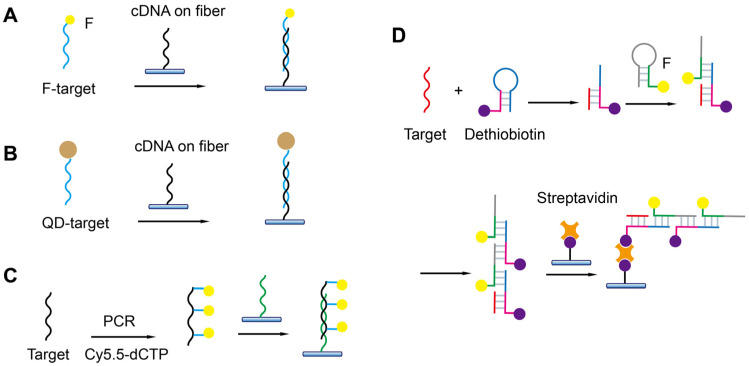
Schematic diagrams of FOEW sensors for detection of nucleic acids via hybridization with (**A**) single-fluorophore Cy5.5-labeled target [107]; (**B**) quantum-dots-labeled target [67]; (**C**) multiple-fluorophore-labeled target synthesized by PCR [108]; or (**D**) multiple-fluorophore-labeled duplex formed via hybridization reaction (HCR) [109].

FOEW sensors have been used for the detection of all types of nucleic acids including DNA and miRNA since its first report in 1995 (Table 3) [103]. In the first demonstration, the 20 mer cDNA probes (dT20) were in situ-synthesized on the surfaces of derivatized quartz optical fibers, and the hybridization on optical fibers was detected by the use of the fluorescent DNA stain ethidium bromide (EB). One year later, a more facile streptavidin–biotin-based strategy similar to what we introduced in Section 4.2 (Figure 5E) was utilized for cDNA immobilization [82]. Different shapes of fibers have been used for the detection of nucleic acids. However, the sensitivity for most FOEW sensors is in the micromolar to low nanomolar range [104], which is too low for practical applications. Therefore, different from the above sensing mechanisms for small-molecule targets and metal ions, the sensing mechanisms for nucleic acids commonly involve signal amplifications, as we summarized below, to improve the sensing sensitivity. The signal amplification strategies can be roughly divided into three categories (Figure 15): (1) using more sensitive optical labels such as gold nanoparticles or quantum dots (QDs); (2) introducing multiple optical labels via synthesis, hybridization chain reaction, or polymerase chain reaction; and (3) other enzymatic reactions. The gold nanoparticles are typically used in the SPR-based FOEW sensors [81,105,106]. In the following, we only summarize the progress of fluorescent FOEW sensors using organic fluorophore or QDs for the detection of nucleic acid targets.

The use of more sensitive optical labels is the easiest way to improve detection sensitivity. For example, the LOD for the detection of Cy5.5-labeled Shigella DNA was 100 pM [107]. In their method, the cDNA was immobilized on the fiber and the signal on detection of target DNA was then realized due to the specific hybridization on the fiber (Figure 15A). The three-strand sandwich detection mode has also been used for the detection of nucleic acid. For example, Zhang et al. realized the detection of miRNA let-7a by using a cDNA probe immobilized on the fiber and a Cy5.5-labeled signal probe complementary to the target (LOD = 24 pM). They also used a “locker” strand to partially hybridize with the target to reduce the false-negative results at the high target concentration [110].

Compared with organic dyes, quantum dots are 20 times brighter and several orders of magnitude more photostable. The LOD was improved to 1 pM when the target was labeled with quantum dots and hybridized with the cDNA on the fiber for 30 min (Figure 15B) [67]. The lower LOD can be achieved by lengthening the hybridization time.

The multiple-fluorophore-labeled target was used for the detection of the microcystin synthetase A gene (LOD = 10 pM) [108]. Cy5.5-labeled deoxycytidine triphosphate (dCTP) was used in the polymerase chain reaction (PCR) to introduce multiple Cy5.5 labels on one target sequence (Figure 15C). The sensitivity was greatly improved by the combination of the CRISPR-Cas13a system and hybridization chain reaction (HCR) (Figure 15D) [109]. Specifically, CRISPR-Cas13a was activated upon binding of the target gene to crRNA. The reporter DNA was subsequently cleaved, and the generated fragment triggered the HCR. The formed duplex contained multiple biotin labels for its immobilization on the fiber and multiple Cy5.5 labels for signal transduction. The LODs of S genes, N genes, and Orf1ab genes were 10, 100, and 10 aM, respectively. The HCR-based multiple Cy5.5 and biotin label strategy has also been used for ultrasensitive detection of miRNA let-7a (LOD = 0.8 fM) [111].

As shown above, all the methods rely on the labeled analyte or intercalation reagents or other reagents for signal amplification and transduction. In 1999, Tan et al. reported the first reagent-free FOEW sensor for the detection of DNA, in which a biotin, fluorophore, and quencher tri-modified molecular beacon DNA probe (MB) was used to replace the cDNA probe. MBs were immobilized on the optical fiber surface via biotin–avidin or biotin–streptavidin interactions. The MBs become fluorescent upon hybridization with target DNA/RNA molecules. The sensor can be used to directly detect, in real-time, target DNA/RNA molecules without using competitive assays. The limitation of the sensor is its low sensitivity (LOD = 1.1 nM) and that the offline PCR amplification of targets is needed. The development of reagent-free FOEW sensors for continuous detections is highly desired, but quite challenging, as we discuss in Section 6.

**Table 3:** Detection of nucleic acids.

Target	Sensing Mechanism	LOD (nM)	Linear Range (nM)	Reusability (Times)	Time (min)	Ref.
DNA	Figure 15B	1 × 10^−3^	0.1–2.5	30	N/A	[67]
Shigella DNA	Figure 15A	0.1	0–2.5	30	5	[107]
dsDNA	N/A	N/A	5 × 10^3^–400 × 10^3^	N/A	0.5	[112]
ssDNA	Figure 15B (AuNP)	0.2 × 10^−3^	N/A	N/A	7	[81]
Let-7a	Fluorescent-labeled signal probes	2.4 × 10^−2^	N/A	N/A	4	[110]
ssDNA	Transmission spectroscopy	10	N/A	N/A	N/A	[104]
Let-7a	Shift in the interference spectrum	0.212	2–2 × 10^4^	N/A	N/A	[113]
Microcystin synthetase A	Figure 15C	10 × 10^−3^	0.05–5	150	7.25	[108]
Let-7a	Figure 15D	0.8 × 10^−6^	1 × 10^−6^–7.1 × 10^−2^	100	N/A	[111]
Let-7a; mRNA 141; let-7c; mRNA 21; mRNA 200	Gold triangular nanoprisms	103 × 10^−9^–261 × 10^−9^	1 × 10^−6^–100	2	N/A	[106]
Three genes of SARS-CoV-2	Figure 15D	10 × 10^−9 a^ 100 × 10^−9 b^ 10 × 10^−9 c^	0–1	100	60	[109]
Chilli Leaf Curl Virus	LSPR of AuNP ^d^	179.3	N/A	N/A	N/A	[114]
Prostate-specific antigen	Figure 15B (AuNP)	0.54 × 10^−6^	N/A	N/A	N/A	[105]

^a^ S, ^b^ N, and ^c^ Orf1ab of SARS-CoV-2; ^d^ local surface plasmon resonance.

### 5.5. Detection of Proteins Using Aptamers

Most biomarkers that are currently tested in clinics are proteins in nature. The major sensing platforms for protein biomarker detection are immunoassays, especially the chemiluminescence immunoassay and electroluminescence immunoassay, due to the high sensitivity, specificity, and reliability. The use of FNA-FOEW sensors for the detection of proteins is rather limited. In most applications, the continuous detection of protein biomarkers may not be necessary. In addition, FOEW sensors have low throughput, which cannot meet the high throughput requirement typically needed for hospital applications. However, an FOEW sensor is a low-cost instrument and is quite suitable for small clinics when the number of samples is not large. The current major effort for the detection of proteins is to improve detection sensitivity. Our group recently isolated several high-affinity DNA aptamers specific to human serum albumin (HSA) [115,116] and used one of the aptamers to construct an NADL-FOEW sensor [50]. The ultrasensitive detection of HSA in urine was realized with an LOD of 0.14 fM. Even though the sensitivity meets the practical requirement, the dynamic range is too broad, which results in a precision issue.

**Figure 16:**
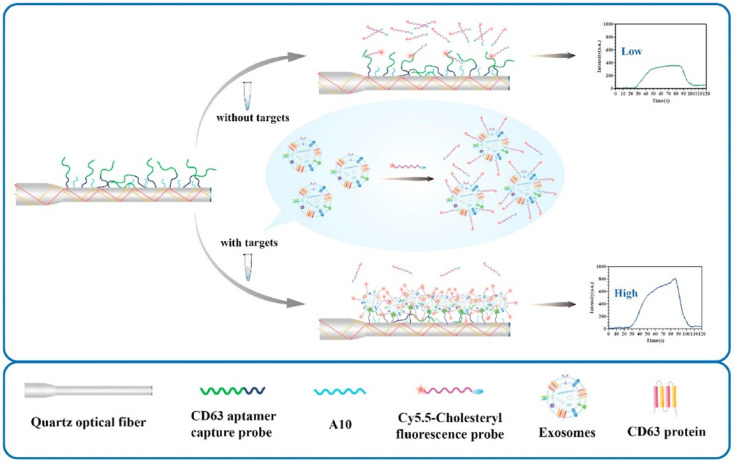
A sandwich-based sensing mechanism for exosome detection using FNA-FOEW sensor. Reprinted from Li et al. (2022) [117], Copyright (2022), with permission from Elsevier.

Exosomes are regarded as a promising biomarker for the noninvasive diagnosis and treatment of diseases. A sandwich-based sensing mechanism was developed for exosome detection (Figure 16) [117]. In their method, an exosome fluorescence probe/aptamer sandwich structure was formed based on both types of interactions: (1) hydrophobic interaction between the cholesteryl on the fluorescent probe and phospholipid bilayer membrane; and (2) CD63 on the surface of the exosome and aptamer attached to the fiber surface. The LOD was 7.66 particles/mL and the linear range was 47.5–4.75 × 10^6^ particles/mL. The detection time was 1 h and the sensor can be regenerated for 60 cycles. The good surface regeneration should be attributed to the surface passivation using BSA.

### 5.6. Detection of Pathogens

Pathogens are microorganisms such as bacteria and viruses that can cause diseases in humans or animals and plants. The development of rapid and low-cost methods for pathogen detection has been a hot topic in recent years. Recent progress on optical biosensors developed for nucleic acid detection related to infectious viral diseases has been recently reviewed [17]. Numerous methods have been reported with a focus on real-time PCR detection kits and immune test strips. Aptamers have also been used for pathogen detections on diverse sensing platforms.

**Figure 17:**
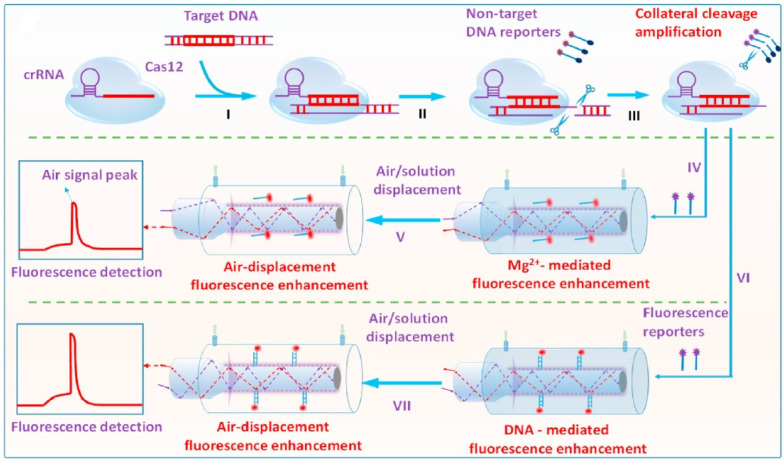
A CRISPR/Cas12a-powered FOEW sensor for nucleic acid amplification-free detection of Staphylococcus aureus with multiple signal enhancements. Reprinted from Song et al. (2023) [120], Copyright (2023), with permission from Elsevier.

Very recently, a few aptamer-FOEW sensors for the detection of pathogens were reported by Long’s group. The detection of *E. coli* O157:H7 was realized by using the fluorophore-labeled aptamer and bare optical fiber (LOD = 610 CFU/mL) [118]. The nonspecific adsorption of the aptamer on the fiber decreased with the increase in the *E. coli* concentration in the sample. They further improved the sensitivity by using a fiber with an in situ etched nanoporous layer to effectively prohibit the entrance of the pathogen-bound Cy5.5 aptamer into the nanopores and to enhance the fluorescence intensity [61]. The LODs for the detection of *E. coli* O157:H7 and *Salmonella typhimurium* were 110 and 210 CFU/mL, respectively. They further realized the simultaneous detection of *E. coli* O157:H7 and *Salmonella typhimurium* using a dual-color FOEW sensor [55]. In their method, the two aptamers were respectively labeled with Cy5.5 and Cy3. The LODs for the detection of *E. coli* O157:H7 and *Salmonella typhimurium* were 340 CFU/mL and 180 CFU/mL, respectively. They also used hybridization chain reaction and CRISPR/Cas12a to detect *E. coli* O157:H7 with an LOD of 17.4 CFU/ mL [119]. An evanescent wave fluorescence nanobiosensing platform based on CRISPR/Cas12a was developed for the ultrasensitive detection of Staphylococcus aureus. (LOD = 13.2 CFU/mL) (Figure 17) [120].

## 6. Challenges and Perspectives

With the improvement of people’s living standard, there is an increasing demand for green environment and food and healthy life. To achieve these goals, intelligent technologies including biosensors are highly desired and the market is rapidly growing. Biosensors with continuous monitoring capability are going to be one of the major types of products for environment protection, food safety, and point-of-care applications. Although some FOEW sensors, such as those used for the measurement of temperature, pH, oxygen, and refractive index, are now commercially available [12], FOEW sensors for the detection of heavy metal ions, small molecules, proteins, nucleic acids, and microorganisms are still at the laboratory research stage. There are some major challenges that need to be overcome to realize the commercial applications of FNA-FOEW sensors.

### 6.1. High-Quality Small-Molecule-Binding Aptamers

Compared to other biosensors, FNA-FOEW sensors are most attractive for the continuous detection of small-molecule targets. One of the major challenges is the availability of high-quality small-molecule-binding aptamers. Even though hundreds of them have been reported in the last 30 years, aptamers with satisfactory affinity (e.g., nanomolar range dissociation constant) and specificity (low crossreactivity required for the detection of a specific target; high crossreactivity for the detection of class-specific targets) are still limited [121]. Aptamers suitable for clinical uses are even rarer due to the presence of numerous structural analogues in vivo. To speed up the discovery of high-quality small-molecule-binding aptamers, the following fields need to be developed first. First, new strategies are needed for the isolation of small-molecule-binding aptamers since the current methods have been demonstrated to be insufficient. Second, crossreactivity tests are either missing or rather limited in many original aptamer isolation papers, which impedes the wide acceptance of aptamers. More systematic characterization of the specificity and crossreactivity is required to push the aptamer field for practical uses. Third, standard affinity characteristic methods are lacking for small-molecule-binding aptamers, causing conflicting results in the literature [122,123]. More general affinity characteristic methods are urgently needed.

### 6.2. Reagent-Free and Continuous Detection

Achieving continuous and reagent-free detection of trace amounts of targets is difficult for biosensors, including FNA-FOEW sensors. Even though extremely low LODs have been achieved by many methods, almost all of them rely on multiple-step signal amplification and/or advanced and expensive instruments. For example, we recently developed NADL-FOEW sensing platforms enabling femtomolar LODs for small-molecule targets without the need for multiple-step signal amplification, but the samples still need to be 1000- to 10,000-fold diluted to minimize the matrix interference. Clearly, the method cannot be used for the direct detection of biomarkers in a reagent-free form like wearable blood sugar electrochemical sensors. To solve this problem, several challenging issues need to be solved. First, the reagent-less detection mode requires target-induced structure switching to realize signal transduction, instead of competitive sensing modes. Aptamers with structure-switching capability are rare, and the rational design of structure-switching aptamers are challenging [124]. Second, continuous and reagent-less detection requires that FNAs can rapidly respond to changes in target concentration. Therefore, both the association and disassociation kinetics need to be fast. However, the study of the kinetic performance of aptamers is quite limited. Third, regenerating the sensor surface without the need for chemicals is quite challenging. So far, the surface regeneration is completely carried out by chemical regeneration [125]. Novel surface modification strategies, especially biomimic layers, are expected to be more effective than the current BSA or nonionic liquid layers. So far, the fundamental studies in this direction are rather limited compared to the development of new sensing mechanisms.

### 6.3. Long-Term Stability under Application Conditions

The long-term stability of FNA-FOEW sensors is critical for continuous detection. Many previous works have demonstrated the successful regeneration of the sensor surface up to 300 times and a storage time of more than one month at 4 °C. However, these tests are typically performed using buffer samples, not real samples, and at low temperatures. The stability tests of FNAs and FNA-FOEW sensors at an outdoor temperature (such as ~40 °C) and in real samples that might contain nucleases have not been reported. These conditions might cause a shortening of the storage time of FNA-functionalized fibers. Modified nucleic acids might be needed to improve the long-term stability and the nuclease-resistance capability.

### 6.4. Throughput

The limitation of the FOEW sensor is its low throughput compared to planar array sensors. Fiber bundles are the future direction to realize the simultaneous detection of multiple targets. The technical challenges are the design and fabrication of the compact optical and signal transduction systems.

## 7. Conclusions

FNA-FOEW sensors have great potential for in situ continuous detection of environmental and food pollutants and biomarkers, with attractive features including low cost, reusability, and portability. We reviewed the recent progress of FNA-FOEW sensors with a focus on the fluorescent FOEW sensors. We sequentially introduced FNAs, the major components of FOEW sensors, and the optical mechanism for real-time fluorescence detection, and the optical fiber interfacial modification methods. The progresses of the sensing mechanisms were then reviewed according to the types of targets, small molecules, heavy metal ions, proteins, nucleic acids, and pathogens. We finally discussed the challenges faced by FNA-FOEW sensors and future directions. FNA-FOEW sensors are a multidisciplinary research field. Technical advances from aptamer isolation, surface modification, organic synthesis chemistry, optics, electrical, and manufacturing engineering are all required. With progressing technical advances, we believe that FNA-FOEW sensors will gradually broaden their application scope from lab benchtop use to onsite practical applications.

## Data Availability

Data are contained within the article.
